# Challenges for the evaluation of digital health solutions—A call for innovative evidence generation approaches

**DOI:** 10.1038/s41746-020-00314-2

**Published:** 2020-08-27

**Authors:** Chaohui Guo, Hutan Ashrafian, Saira Ghafur, Gianluca Fontana, Clarissa Gardner, Matthew Prime

**Affiliations:** 1 Roche Diagnostics, Basel, Switzerland; 2 Imperial College London, London, UK

**Keywords:** Health policy, Translational research

## Abstract

The field of digital health, and its meaning, has evolved rapidly over the last 20 years. For this article we followed the most recent definition provided by FDA in 2020. Emerging solutions offers tremendous potential to positively transform the healthcare sector. Despite the growing number of applications, however, the evolution of methodologies to perform timely, cost-effective and robust evaluations have not kept pace. It remains an industry-wide challenge to provide credible evidence, therefore, hindering wider adoption. Conventional methodologies, such as clinical trials, have seldom been applied and more pragmatic approaches are needed. In response, several academic centers such as researchers from the Institute of Global Health Innovation at Imperial College London have initiated a digital health clinical simulation test bed to explore new approaches for evidence gathering relevant to solution type and maturity. The aim of this article is to: (1) Review current research approaches and discuss their limitations; (2) Discuss challenges faced by different stakeholders in undertaking evaluations; and (3) Call for new approaches to facilitate the safe and responsible growth of the digital health sector.

## Introduction

Digital health has evolved rapidly since the concept was first introduced in 2000 by Seth Frank^1,2^. The FDA considers digital health as a broad scope that includes categories such as mobile health, health information technology, wearable devices, telehealth and telemedicine, and personalized medicine^3^, a definition we follow in this article. Indeed, the numbers of digital health solutions are booming, for example, more than 300,000 health applications exist with more than 200 added daily^4^. Digital solutions can be grouped as follows, based on potential risk to patients^5^: (1) Solutions that improve system efficiency but with no measurable patient outcome benefit; (2) Mobile digital health, that inform or deliver basic monitoring, and encourage behavior change and self-management; (3) Clinical decision support (CDS), and prediction models, that guide treatment, deliver active monitoring, calculate and/or diagnose.

The evidence requirements of regulators are determined by a product’s intended use claims, as such, a large proportion of digital health solutions (e.g. administrative tools and wellness apps) fall outside of their jurisdiction. Therefore, a huge challenge for end users, such as patients and providers (e.g. healthcare professionals, hospital administrators), is how to determine a new solution’s credibility and compliance with standards. Furthermore, end users have different thresholds for acceptance of innovation and can be grouped into five archetypes: innovators, early adopters, early majority, late majority, and laggards^6^. In addition, aging adults, considered amongst the most digitally divided demographic group^7^, present unique challenges and dedicated efforts exist to develop strategies for implementation^7–10^. Conversely, challenges exist for healthcare innovators to best demonstrate solution impacts and to ensure compliance with standards, these include: unclear end-user expectations; uncertainty of evidence generation approaches; and, keeping up to date with the evolving compliance landscapes.

This article discusses the challenges for providing timely and robust evidence, to meet end-user expectations, in the context of digital health solutions. Specifically, we consider how the cadence of traditional research approaches are misaligned with the “fail fast, fail often” mantra espoused by technology start-ups. In addition, we introduce clinical simulation-based research as a potential opportunity to bridge the evidence gap.

## A rapidly evolving guidance and regulatory landscape

Over the last 10 years a plethora of guidance has been developed for digital health innovators. In Table 1, we highlighted 10 of the key guidance (e.g., Continua Design Guidelines 2010, WHO monitoring and evaluating digital health solutions 2016, NICE evidence standards framework 2019; US FDA pre-certification program—a working model 2019, and FDA Proposed Regulatory Framework for modifications to Artificial intelligence/Machine learning-based Software as a Medical Device 2019). We ordered them by date first published and provided for each guidance a brief summary, applicable areas within digital health, releasing organization, and its main activities (Table 1). We observed that development of such documents follows a pattern: initial development by industry, optimization by non-government organizations, and finally refinement by government agencies. In addition, academic initiatives and institutions have produced critical thought leadership, often acting as counterbalance to industry proposals (Table 2; The digital health scorecard 2019). In Table 2, we highlighted five academic recommendations relevant to undertaking evidence generation studies for digital health solutions.

**Table 1:** Selected guidance and discussion documents relevant to digital health solutions (not exhaustive).

Document title	Document descriptions	Applicable areas within digital health	Date first published	Organization responsible	Main activities of releasing organization
Continua Design Guidelines (CDG)^97^	Defining a framework of underlying standards and criteria that are required to ensure the interoperability of components used for applications monitoring personal health and wellness.	Connected devices and mobile applications (particular emphasis on interoperability and data standards)	2010	Personalized Connected Healthcare Alliance (PCHA)	PCHA is a membership-based Healthcare Information and Management Systems Society (HIMSS) Innovation Company. HIMSS is a global advisor and thought leader supporting the transformation of health through the application of information and technology.
FDA’s benefit-risk framework for medical devices^11^	Providing a general framework to evaluate medical devices in both the benefits (7 dimensions, such as types, magnitude, likelihood of patients experiencing one or more benefits, etc.) and risks (7 dimensions, such as risk severity, likelihood of risk, false-positive or false-negative results, patient tolerance of risk, etc.)	All digital health solutions	2016	Food & Drug Administration (FDA)	The FDA is responsible for protecting and promoting public health throught the control and supervision, amongst other things, e.g., medical devices.
WHO monitoring and evaluating digital health interventions^18^	Provides a general framework for the evaluation and validation of digital solutions along its product mature life-cycle	All digital health solutions	2016	World Health Organization (WHO)	International Public Health
Guidelines on the Qualification and Classification of Stand Alone Software Used in Healthcare within the Regulatory Framework of Medical Devices (MEDDEV 2.1/6)^98^	Describing when software does and does not qualify as a medical or in vitro diagnostic device	Healthare software	2016	European Commission	Executive branch of the European Union
IMDRF SaMD Key Definitions (N10), IMDRF SaMD Risk Categorization Framework (N12), IMDRF SaMD Quality Management Systems (N23), and IMDRF SaMD Clinical Evaluation (N41)^99^	Documents developed by the International Medical Device Regulators Forum (IMDRF) as the basis for SaMD regulatory efforts in various countries	Software as a Medical Device (SaMD)	2017	International Medical Device Regulators Forum (IMDRF)	The International Medical Device Regulators Forum (IMDRF) was conceived in February 2011 as a forum to discuss future directions in medical device regulatory harmonization. It is a voluntary group of medical device regulators from around the world who have come together to build on the strong foundational work of the Global Harmonization Task Force on Medical Devices (GHTF), and to accelerate international medical device regulatory harmonization and convergence.
US FDA mobile medical apps guidance^12^	Risk-based approach (not all mobile apps are subject to FDA regulation). The agency oversees most mobile apps that are intended to treat, diagnose, cure, mitigate, or prevent disease or other conditions as medical devices under federal statute.	Mobile medical applications	2018	Food & Drug Administration (FDA)	The FDA is responsible for protecting and promoting public health throught the control and supervision, amongst other things, e.g., medical devices.
Code of conduct for data-driven health and care technology^13^	Provides 10 principles for developing and evaluating digital health solutions	All digital health solutions	2019	UK Department of Health & Social Care	Responsible for policy on health and social care matters in England & Wales
NICE evidence standards framework^5^	Providing guidance on evidence generation for effectiveness standards (section A) and economic standards (section B). Guiding the user through identifying the functional classification of their product (three-tier based on risk to users). For products with higher tier functions, the gold standard for evaluating their effectiveness is a high-quality intervention study or randomized controlled trial (RCT).	Digital health solutions (excl. products with adaptive artificial intelligence algorithms)	2019	National Institute for Health and Care Excellence (NICE)	Executive non-departmental public body of the Department of Health in the United Kingdom which publishes guidelines, amongst other things, the use of health technologies within the National Health Service (NHS)/
US FDA pre-certification program (a working model)^14^	Provides a voluntary pathway for efficient oversight of software-based medical devices from manufacturers who have demonstrated a robust culture of quality and organizational excellence (CQOE) and are committed to monitoring real-world performance	Low risk digital health solutions	2019	Food & Drug Administration (FDA)	The FDA is responsible for protecting and promoting public health throught the control and supervision, amongst other things, e.g., medical devices.
Proposed Regulatory Framework for modifications to Artificial intelligence/Machine learning-based Software as a Medical Device (SaMD)^100^	Proposed framework for modifications to AI/ML- based SaMD that is based on the internationally harmonized International Medical Device Regulators Forum (IMDRF) risk categorization principles, FDA’s benefit-risk framework, risk management principles in the software modifications guidance, and the organization-based TPLC approach as envisioned in the Digital Health Software Precertification (Pre-Cert) Program. It also leverages practices from current premarket programs, including the 510(k), De Novo, and PMA pathways	AI and ML algorithms-based solutions	2019 (draft document released for feedback)	Food & Drug Administration (FDA)	The FDA is responsible for protecting and promoting public health throught the control and supervision, amongst other things, e.g., medical devices.

**Table 2:** Selected academic recommendations relevant to undertaking evidence generation studies for digital health solutions (not exhaustive).

Tool/framework	Document descriptions	Applicable areas within digital health	Date first published
Quality in prognosis studies (QUIPS)^101^	6 factors to consider when evaluating validity and bias in studies of prognostic factors: participation, attrition, prognostic factor measurement, confounding measurement and account, outcome measurement, and analysis and reporting	Prognosis models (incl., individualized predictive model)	2006
The Cochrane risk-of-bias tool for randomized trials (RoB2)^102^	Set of domains of bias to guide the evaluation about features of a trial that are relevant to risk of bias based on answers to the signaling questions	Randomized studies (suitable for individually randomized, parallel-group trials)	2008 (updated in 2011)
The risk of bias in nonrandomized studies of interventions (ROBINS-I)^103^	Tool to assess risk of bias in non-randomized studies over 7 domains (e.g., missing data, participant selection, etc.)	Non-randomized studies	2016
PROBAST: A Tool to Assess the Risk of Bias and Applicability of Prediction Model Studies^104^	Tool to assess the risk of bias and applicability of prediction model studies (20 questions). Informed by a Delphi procedure involving 38 experts and refined through piloting. It is not suitable for comparative studies.	Predictive models (incl., CDS algorithms)	2019
The digital health scorecard^2^	Academic developed framework that proposes validation should include three aspects: (1) technical validation (e.g., how accurately does the solution measure what it claims?), (2) clinical validation (e.g., does the solution have any support for improving condition-specific outcomes?), (3) system validation (e.g., does the solution integrate into patients’ lives, provider workflows, and healthcare systems).	All digital health solutions	2019

Until recently regulators relied upon modifications to existing medical device (software) regulations and innovators were encouraged to conform to development standards, as shown in Table 3, where we highlighted eight regulations and standards relevant to digital health solutions (e.g., IEC Medical device software, ISO Health informatics—requirements for an electronic health record architecture). However, the speed of development, diversity of interventions, and potential risks has finally prompted policy-makers to produce more targeted guidance on solution classification and evidence requirements^5,11–14^ (Tables 1 and 3). For example, one initiative, the FDA Pre-certification Program^14^, seeks to streamline the approval of Software as a Medical Device (SAMD), and proposes to assess both development organization and product capabilities. Notwithstanding, current guidance does not go far enough to enable innovators and end-users to know what evidence generation approaches are appropriate, and practical, for all classes of digital health solutions throughout the product lifecycle.

**Table 3:** Selected regulations and standards relevant to digital health solutions (not exhaustive).

Document title	Document descriptions	Applicable areas within digital health	Date first published	Organization responsible	Main activities of releasing organization
IEC 62304: Medical device software– software life cycle processes^105^	Providing life cycle requirements for the development of medical software and software within medical devices. It is harmonized by the European Union (EU) and the United States (US)	Medical device software	2006	International Standards Organization (ISO)	International Electrotechnical Commission
ISO 18308:2011 Health informatics— Requirements for an electronic health record architecture^106^	Defines the set of requirements for the architecture of a system that processes, manages and communicates electronic health record (EHR) information: an EHR architecture	IT applications in healthcare technology	2011	International Standards Organization (ISO)	The International Organization for Standardization is an international standard-setting body composed of representatives from various national standards organizations. ISO (35.240.80) pertains to IT Applications in Health Care Technology
ISO/TR 12300:2014 Health informatics— Principles of mapping between terminological systems^107^	Provides guidance for organizations charged with creating or applying maps to meet their business needs.	IT applications in healthcare technology	2014	International Standards Organization (ISO)	As above
ISO/HL7 10781:2015 [HL7] Health Informatics— HL7 Electronic Health Records-System Functional Model, Release 2 (EHR FM)^108^	Provides a reference list of functions that may be present in an Electronic Health Record System (EHR-S)	IT applications in healthcare technology	2015	International Standards Organization (ISO)	As above
US 21st Century Cures Act^109^	Providing broad scope related to health, with one part covering expedited product development programs, including the regenerative medicine advanced therapy, and the breakthrough devices program (designed to speed the review of certain innovative medical devices)	All digital health solutions	2016	Passed by Congress and signed into law by the President	NA
Medical Device Regulation (MDR)— Regulation (EU) 2017/745^110^	The entirety of the Regulation is applicable for SaMD products, however, classification Rule 17 specifically applies to software products	Software as a Medical Device (SaMD) (Rule 17)	2017	European Commission	Executive branch of the European Union
ISO 25237:2017 Health informatics— Pseudonymization^111^	Contains principles and requirements for privacy protection using pseudonymization services for the protection of personal health information.	IT applications in healthcare technology	2017	International Standards Organization (ISO)	As above
ISO/IEC CD 23053 Framework for Artificial Intelligence (AI) Systems Using Machine Learning (ML)^112^	Under development	IT applications in healthcare technology	Upcoming	International Standards Organization (ISO)	As above

## Traditional approaches to evaluation of digital health solutions

**Fig. 1:**
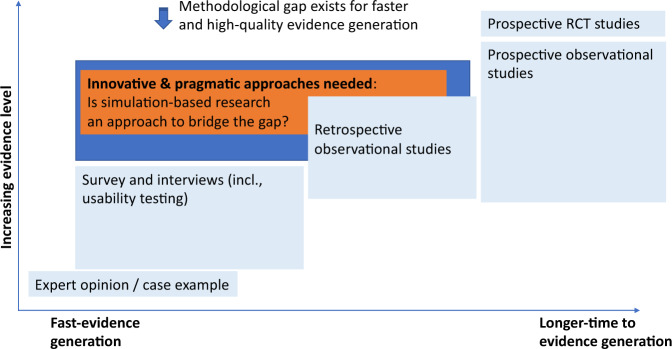
Existing approaches for health digital solution evaluation, current methodological gap and emerging innovative pragmatic approaches to fill such gap. Note, the position of each methodology is meant to be illustrative and reflecting general cases.

The most commonly recognized evidence for healthcare interventions is the randomized controlled clinical trial (RCT)^15,16^, yet, only a handful of products have been tested in this way as shown by recent systematic review^17^ and our searching results in Table 4, where we illustrated recent studies evaluating digital solutions and their methods (including study designs, study length, sample size, etc.). Indeed, a recent systematic review of publications between 1995 and 2016 identified just 24 RCTs for the high-risk CDS category^17^. In our opinion, this lack of studies indicates that these methods are no longer practicable, likely due to the speed of digital product development and iterative upgrading. In Fig. 1, we mapped existing approaches along two dimensions; strength of evidence and study duration, which demonstrated the current methodological gap to evidence needs and opportunity for more innovative and agile approaches. In this section we highlight a few of the more common methodologies, discuss strengths and limitations, and provide examples of their application (Table 4).

**Table 4:** Recent studies utilizing various methodologies in evaluating digital health solutions (not exhaustive).

Evaluation approach	Digital solution	Design/method details	Sample size	Number of sites involved during evaluation	Study length
Prospective: Randamized comparative design	Web-mediated follow-up algorithm^113^	A web-mediated follow-up algorithm based on self-reported symptoms improved OS (18 vs. 12 months in the experimental and control arm, respectively) due to early relapse detection and better performance status at relapse	120 patients	Single	2 years (2014–2016)
	Patient-reported outcomes collected via tablet^36^	Patients randomly assigned to routine outpatient chemotherapy for advanced solid tumors with patient-reported outcomes vs. usual care with symptom monitoring at the discretion of clinicians	766 patients, with 441 in the intervention cofthagenondition and 325 in the control condition (usual care)	Single	4 years (2007–2011)
	Text messaging^37^	Randomized trial of text messaging to reduce early discontinuation of aromatase inhibitor therapy in women with breast cancer	338 patients on Text messaging (TM) condition, and 338 on non-TM	Multi	3 years
	Digitally enabled care pathway for acute kidney injury management^114^	Clinical outcome data were collected from adults with AKI on emergency admission before and after deployment at the intervention site and another not receiving the intervention (multi-site, pre- & post intervention design)	Implementation site: 767 patients in pre vs. 439 in post; Control site: 1016 in pre- and 422 in post	Multi	2 years (2016–2017)
	WellDoc® mobile diabetes management tool^38^	Patients with type 2 diabetes were recruited from three community physician practices and evenly randomized between intervention (cell phone-based software designed by endocrinologists, etc.) and control (One Touch Ultra™ BG meters)	13 patients with type 2 diabetes in the intervention group vs. 13 in control	Multi	End-2-end study length not found in the paper
Prospective: custer randomized design	Internet-delivered pain self-management program (WebMAP)^115^	Protocol proposal for employing a stepped wedge design in which the WebMAP mobile intervention is sequentially implemented in 8 specialty pain clinics following a usual care period	120 children to be recruited	Multi	NA
	Telehealth programs^41^	A Cluster-Randomized Program Evaluation in the Veterans Health Administration to evaluate the impact of availability of Telehealth Programs on Documented HIV Viral Suppression	Immediate telehealth availability (*n* = 925 patients in service areas of 13 primary care clinics offering telehealth) vs. availability 1 year later (*n* = 745 patients in 12 clinics)	Multi	2015–2016
	Digital medicine offering (DMO; medication taken with ingestible sensor) measuring medication ingestion adherence, physical activity, etc.^116^	Participants with elevated systolic BP (SBP ≥ 140 mm Hg) and HbA1c (≥7%) failing antihypertensive (≥2 medications) and oral diabetes therapy were enrolled in this three-arm, 12-week, cluster-randomized study	109 participants (12 sites) in total, within 80 participants (7 sites) in the DMO condition, and 29 participants (5 sites) in usual care	Multi	12 weeks for the conduction stage; end-to-end study length not reported
Prospective: Micro-randomization design	HeartSteps, an mHealth intervention that encourages regular walking via activity suggestions tailored to the individuals’ current context^52^	A micro-randomized trial to evaluate the efficacy of HeartSteps’ activity suggestions to optimize the intervention; Contextually tailored suggestions could be delivered up to five times per day at user-selected times, for each participant on each day of the study, HeartSteps randomized whether to provide an activity suggestion, and, if so, whether to provide a walking or an antisedentary suggestion	44 adults were recruited	Single	Recruitment took place from August 2015 to January 2016; study conduction took 6 weeks; in total it took 4 years from recruitmnt (2015) to publishment (2019)
Prospective: Pre–post intervention design	Clinical decision support system to aid computerized physician order entry of chemotherapy order (C-CO)^117^	Computerized chemotherpay order (C-CO) versus paper based chemotherapy order (P-CO) in a 30-bed chemotherapy bay of a tertiary hospital.	9279 chemotherapy orders from patients	Single	Not reported
	Computerized provider order entry (CPOE)^118^	CPOE system was implemented throughout the hospital, and impacts (e.g., active order number, medication-related patient safety events) were measured	212 medication-related events	Single	Not reported
Prospective: computational simulation	A deep-learning framework (Med3R) as clinical decision support^119^	Evaluating a deep-learning framework (Med3R), which utilizes a human-like learning and reasoning process, its performance was measured against general human examinees and similar leading product (i.e., WatsonQA system) in mocked written test of National Medical Licensing Examination in China	Before officially taking National Medical Licence Examination for China (NMLEC) 7 practice tests were undertaken by Med3R to evaluate performance. In 2017 the Med3R system was officially entered as a “special examineee” and succesfully passed with a score of 78%.	Multi	Not reported
	Direct order entry by physicians into computer-based medical information systems^81^	A computer simulation model was developed to represent the process through which medical orders are entered into a digital hospital information system and estimate the impacts on process improvement, reduction of errors, and improved communication etc.	No participant was involved for real-time; instead, four weeks of patient data were extracted from the information system; 227 simulations of order entry were conducted	Single	Simulation itself took 16 h; end-to-end timeline not reported
Prospective: Clinical simulation	A CDS tool that embedded clinical prediction rules into primary care workflow in EMR systems^90^	Physicians interacting with video clips of standardized trained patient actors enacting the clinical scenarios (Pneumonia and Strep cases)	8 (3 resident and 5 faculty providers) in the clinical simulation phase	Single	Not reported
	Enhanced electronic health records system with features such as automatic sorting and decision support instructions^91^	Physicians randomly assigned to baseline EHR or enhanced EHR; cognitive load for physicians and performMazurance were evaluated for each condition	38 (20 in baseline, 18 in intervention)	Single	9 months (2016 April–Dec)
	Medication management system known as “Patient Safety through Intelligent Procedure in Medication” (PSIP-DK)^120^	Physicians randomly assigned to baseline (local standard mangement system) or the new system (PSIP-DK); participating doctors were asked to perform a ward round on the five patients; impact on medical safefy were evaluated based on semi-structural interviews	15 (10 doctors and 5 patients) and 50 simulation runs—25 using PSIP-DK and 25 using standard system	Single	6+ months
	CDSS with weight-loss prediction model^121^	Physicians with varying experience levels were then recruited to evaluate 100 patients in an independent validation data set of head and neck cancer twice (i.e., a pre-post design)	4 physicians evaluating 100 patient cases	Single	Not reported
	Voice assistants (recognition of commonly dispensed medications)^122^	Voice recordings of 46 participants were played to voice assistants (e.g., Alexa, google assitant, siri) and compare the recognition accuracy rates	46 participants (voice recorded as stimuli)	Single	2+ months (All voice recordings were analyzed between mid-December 2018 and mid-January 2019—end-to-end study length not reported)
Retrospective (incl. hybrid with prospective)	Watson for Oncology^67^	Treatment recommendations made by WFO (638 breast cancers) and the tumor board were compared to evaluate the concordance in hospitals of India	638 breast cancer patients	Not applicable/reported	2 years (2016–2018)
	Electronic symptom screening assessment scale (ESAS)^123^	Retrospective chart reviews on cancer patient visits at a regional cancer centeremote and/or distributedr to examine whether patient visits with higher ESAS symptom scores are associated with higher rates of symptom documented in the chart and symptom-specific actions being taken	912 visits were identified	Single	Study length not reported

### Surveys and interviews

In the early stages of development innovators seek to establish product usability, feasibility, and efficacy^18^. Surveys and/or interviews are often employed, which are low-cost, efficient, scalable tools to collect attitudes, user experience, and suitability insights. Commonly used methods include usability testing, user-center design, net promoter score survey (e.g. to rate likelihood to recommend a product), online surveys, and log-file data analyses (e.g. to evaluate how users interact with the digital solution)^19^. Such approaches have been used to explore user views on the usefulness of digital storytelling^20^, to assess a web-based network for MS patients^21^, and to collect attitudes towards digital treatment for depression^22^. Despite being common, few efforts are turned into peer-reviewed publications^19^, likely because the main purpose was to generate insights for internal use (e.g. product development) or external customer communication (e.g. case studies, presentations), and can be challenging to pass the peer-review for such work due to its relatively lower evidence strength^19,23^.

A key approach for digital solution development is usability testing which has been widely utilized to examine whether specified users can achieve intended use effectively and efficiently^24–26^. Typically, an intended user completes tasks and is observed for where they encounter problems. This can be exploratory, to identify new features or functionalities, or comparative testing A vs. B^27,28^. Studies are conducted by UX researchers, who synthesize results and translate to actions (e.g. product improvements). Data collected can be qualitative (e.g. observations of problems) and/or quantitative (e.g. task time, task success rates). Evidence strength depends upon study design, for example, task-based and controlled studies that collect quantitative data and can be replicated in other settings/sites, generate stronger evidence, whilst surveys and self-reported behaviors provide weaker evidence, as suggested by UX practitioners^29^. Controversy exists regarding the appropriate number of participants. Whilst there is no “single correct number”, for formative testing 5 participants is common (“the magic number 5”), compared with 20 participants for summative tests, which offer a tighter confidence interval^30^.

### Prospective studies

Prospective RCTs are the most accepted method for evaluating healthcare interventions^31^. For end-users, not considered “early adopters”, such studies are critical to justify adoption decisions. The randomization unit can be individuals, groups (“clusters”), or even specific solution components^32^. Choice of the study designs heavily depends on the digital solution and objectives of the evaluation.

Individual-randomization trials (IRTs) are well-suited for digital solutions targeting an individual user, such as patient-level randomization (e.g. symptom self-monitoring^33^) or clinician-level randomization (e.g. digital pathology algorithms for pathologists^34^). This is traditionally the most commonly used experimental design in healthcare research (e.g., clinical trials for the development of drugs and diagnostic tests)^35^, however for digital health solutions, we found few studies employed strict individual randomized designs (Table 4; e.g., refs. ^36–38^). One reason is that individual randomization is not always possible or appropriate as in the examples provided below.

Cluster-randomization trials (CRTs), by contrast, are better suited for digital solutions supporting group efforts (e.g. solutions supporting tumor board meetings^39^), and this approach has been increasingly adopted by public health researchers^40–42^. CRTs are often used in situations when contamination may occur; for example, where individuals in the same cluster have been randomized to different intervention groups, or for logistic, feasibility or ethical reasons^43^. Attractive features include: increased administrative efficiency; decreased risk of experimental contamination (e.g. where control group individuals adopt the intervention)^43^; and, enhancement of subject compliance^44^. In addition, CRTs allow both direct and indirect effects of an intervention to be evaluated—a particular advantage when both effects are hypothesized to be important, e.g., in vaccine field trials^45^. Disadvantages include: reduced statistical efficiency relative to IRTs^46^; overmatching; and, subsampling bias^47,48^. Analysis commonly employs multi-level modeling^49,50^.

Micro-randomization trials (MRTs) are helpful when researchers want to determine empirically the efficacy of a specific component (e.g., which component of an intervention should be delivered, and whether it had the intended effect)^32^. MRT involves randomly assigning an intervention option at each time point that the component could be delivered (e.g., see examples in the ref. ^51^ on p. 5 and ref. ^52^)^51,52^, and can be particularly powerful in the early stages of product development^51^. MRTs generate longitudinal data with repeated measures of participants’ behaviors, context, and psychosocial factors, and can be analyzed by methods, such as multilevel models and generalized estimating equation^51,53,54^.

The most commonly used method for evaluating digital health solutions, however, is the pre–post design, as demonstrated by a previous systematic review^17^ and supported by our own searches (Table 4). A standard approach of pre–post design involves: pre-phase, which provides control data; “washout” period^55^ (i.e., with no interventions implemented with a time gap up to several months), to allow familiarization and to limit bias related to implementation^39,56^; post-phase to collect data on solution effectiveness. Existing studies are often undertaken at a single site (vs. multi-site), which is typically more practical and affordable. Typically, this design requires a longer duration, making it difficult to evaluate continuous solution upgrades (i.e. new features and/or bug fixes), which are often observed in digital health products. In addition, it is not optimal for testing medium-term or longer-term clinical outcomes, because it is difficult to determine independent effects when patients may appear in both pre-phase and post-phase. Data analysis generally employs methods, such as analysis of variance (ANOVA) and analysis of covariance (ANCOVA) or non-parametric tests (depending on the underlying distributions)^57^.

Relatively few multi-site studies have been conducted^17^ (we also listed some examples in Table 4), nevertheless, a variety of designs have been attempted in this context including: pre–post^58^, cross-sectional with non-equivalent control^59^, cross-sectional with internal control^60^, and randomized controlled trial^61^. For multi-site RCTs, some sites are assigned as controls and the rest as the experimental condition. For this approach, control and experimental sites should be matched along key characteristics (e.g., workflow, patient characteristics), which can be difficult to achieve. The main advantage is reduction in study duration. Disadvantages include: higher set-up efforts; increased cost; and, challenges to identify matched sites. Various tests are employed such as *t*-test, non-parametric tests, or other advanced techniques (depending on the underlying distributions)^62^.

### Retrospective studies

Retrospective studies can be employed to analyze pre-existing data, such as patient charts or electronic medical records. Types of retrospective studies include case series, cohort, or case-control studies. They are typically quicker, cheaper, and easier^63^ than prospective studies because data are already collected, and are commonly used to generate hypotheses for further investigation by prospective studies. The disadvantages are, that they are subject to biases and confounding factors, such as patient information loss or distortion during data collection^64^, risk factors present but not captured, normal growth or maturation influence, attrition bias (e.g. patients with unfavorable outcome(s) less likely to attend follow-up)^63,65^, and selection bias due to non-random assignment of participants^65,66^. Such biases threaten internal validity, therefore, retrospective studies are considered (particularly by the academic groups) inferior as compared to RCTs^63–66^. It remains as an open question whether this is still the case for digital health solutions, particularly for the ones of lower-risk class.

To date, few publications have evaluated digital solutions with retrospective data, likely due to limited use of digital solutions in clinical practice, and challenges for data access (e.g. GDPR). Nevertheless, one such study from India investigated concordance between the treatment recommendations of an artificial intelligence (AI) algorithm compared with actual tumor board recommendations^67^ (Table 4). Strictly speaking this study was a hybrid of retrospective (treatment recommendations from Tumor Board 2014–2016) and prospective (treatment recommendations from AI algorithm in 2016). A key limitation of the study was that breast cancer treatment knowledge was not constant for the two conditions, because of the evolving clinical practice standards. Additional, prospective studies would be required to examine impacts on clinical outcomes, efficiency, and mental fatigue of clinicians.

### Systematic reviews

Systematic reviews have a key role in evidence-based medicine and the development of clinical guidelines^68–70^. Reviews on a specific solution can provide stronger evidence for its impacts, but require a sufficient number of individual evaluation studies. A possible limitation for such work in digital health is that included studies would need to be matched to the same mechanism of intervention, disease area, and measurable outcome.

Systematic reviews of prediction models are a new and evolving area and are increasingly undertaken to systematically identify, appraise, and summarize evidence on the performance of prediction models^71–73^. Frameworks and tools exist to facilitate this including: prediction model risk of bias assessment tool (PROBAST), quality in prognosis studies (QUIPS), revised Cochrane randomized comparative design (ROB), risk of bias in nonrandomized studies of interventions (ROBINS-I). Details provided in Table 2.

### Economic evaluation

Demonstration of positive economic benefits are critical for the majority of end-users to justify solution adoption. In addition, such data is important for other critical actors (e.g. Payers, Government agencies, Professional Societies) to endorse the need for change. The World Health Organization (WHO) guidelines provide a good overview of options for economic evaluation (Table 4.8 in WHO guideline^18^) including: cost-effectiveness analysis, cost–benefit analysis, cost-consequence analysis, cost-minimization analysis, etc. However, for all of the aforementioned methods, tracking usage and performance data of users compared to non-users, is required.

### The critical evidence gaps for digital health solutions

In general, approaches for evidence generation at early stages of product development deliver weaker evidence. Although, such efforts may be enough to support internal needs, and can convince “early adopters”, they are insufficient to satisfy the “majority” of a solution’s potential beneficiaries. These groups require, and expect, more robust, traditional evidence approaches. Currently, and in our opinion, there is a gap between quick, lower-cost approaches applied at the early stages of product development and higher-cost approaches needed to convince the majority of stakeholders.

## The challenge of the traditional approach for digital health innovators

It is our opinion that traditional methods to develop more robust evidence are incongruent with the agile approach taken in software development (e.g., mismatch between the length of RCTs and the typical development and update cycle of software). As such, traditional approaches present fundamental limitations for researchers to create evidence for digital health solutions. In fact, evaluation of digital health solutions has been identified as requiring improvement, and has been cited as a major obstacle for wider adoption^74–76^. The paradox at the heart of this problem is that, “without evidence healthcare providers would not adopt a solution; without solution adoption it is very difficult to generate evidence to convince healthcare providers”.

Digital solution evaluation requires collective efforts from multiple parties, such as health authorities, healthcare providers (incl., academic medical centers), and manufacturers such as small and medium-sized enterprises (SMEs), multinational corporation (MNCs). Whilst they face shared difficulties with the current approaches for evidence generation (e.g. significant time and cost), they also have circumstance-specific challenges.

### SMEs—Limited resources to undertake clinical studies

SMEs typically prioritize and allocate their research and development budget to product development. Anecdotal evidence suggests that close relationships between innovator and adopter are a critical driver of initial adoption decisions. Wider implementation requires robust evidence of benefit, yet this is difficult to prioritize given the many challenges for establishing new ventures. In addition, well designed and executed studies require skilled researchers, often via collaboration with academia, adding further complexity. Moreover, it has been estimated that the timescale for submitting a research proposal and receiving ethical approval for a pilot or trial study can take as long as 3 years^19^. As demonstrated in a recent report^19^, the biggest obstacle for providing evidence of effectiveness reported by companies, is the cost and timeframe for evaluation.

### MNCs—Out of date evidence not an investment priority

Larger corporations have more resources to develop evidence but are equally limited by time. For internal budget allocation, it can be difficult to provide rationale for investments into expensive and time-consuming clinical studies for early-stage solutions when such products are constantly evolving. Given it typically takes 2–3 years to conduct a study, evidence published today may reflect a product that has been updated and refined multiple times. Furthermore, for many companies’ investments in sales and manufacturing, for example, are more tangible with more predictable return on investment than those in clinical studies.

The same challenges (as SMEs) exist around navigating the complex infrastructure of the healthcare system, dealing with the cultural resistance to digital solutions, and identifying appropriate principle investigators for the evaluation studies. Despite the long-existing collaborations between large health abd life science companies and principal investigators in, for example clinical trials for drug development, this group of researchers may not necessarily be willing to conduct studies to evaluate digital solutions, as they require different settings, capabilities and also deliver different scientific output—benefits on the operational level impacting cost and indirectly patient outcome versus a drug that can improve patient outcome directly.

### Academic institutions—focus on research output not widespread adoption

A growing number of academic centers have created digital health research programs to develop and evaluate digital health solutions. However, such research units generally favor traditional research methodologies because of the increased likelihood of high-impact publication. As such, the timeliness of studies is largely immaterial, therefore, potentially valuable solutions may be delayed and/or are never implemented at scale. Obtaining sufficient research funding can also be a challenge.

## Evolving pragmatic approaches for evidence generation

In our opinion, large differences exist between the evidence required for initial adopters (e.g., surveys and interviews, case studies), and that required for the majority (prospective RCT studies). Other research areas, such as drug development, have demonstrated that pragmatic approaches can be adopted to control cost at early stages (pragmatic clinical trials, basket of baskets, umbrella trials, etc.^77–79^). The “gold standard” RCT remains but for later-stage final assessment.

The concept of “simulation” is not new and is the methodological foundation for human behavior experimental research (e.g. neuroscience and experimental psychology). The assumption is that people behave similar to real-life if key components of the scenarios are extracted and fidelity maintained. Various approaches for simulation could be applied to evaluate digital solutions, such as, computational, system, and clinical simulation.

Computational simulation for software evaluation involves two steps: verification and validation^80^. Verification checks if a system was built according to specification, and validation checks that a system meets user expectations. The most common application of computational has been for verification. Typically, this involves simulated outcomes based on synthesized or real cases, before involving users/clinicians. Recent efforts have extended its use to non-regulated and on-market products (e.g., Google Alexa; Table 4). This approach is more applicable for products where the outputs can be evaluated for individual users, and not for clinical management tools where a group of users are targeted (e.g. multidisciplinary tumor boards).

System simulation adopts a system engineering view and methodology to model the effect of an intervention on a healthcare system (e.g. multi-site hospital network) without disrupting the real health care setting^81^. It has gained some traction (ASCO QCS Keynote topic by Joe Simone, literatures^82,83^), however, to date we are not aware of the use of system simulation to evaluate a digital health solution, perhaps because of the significant complexity to establish models that represent a healthcare system.

Clinical simulation was traditionally developed and used in training medical residents, and it was further developed as an approach to test systems and digital solutions with representative users doing representative tasks, in representative settings/environments^84^. In our opinion, can be complementary to many of the traditional approaches reviewed above that require the use of a digital solution in real clinical practice, and could bridge the evidence needs between those of “early adopters” and the “majority”. Clinical simulation provides a good balance between the strength of evidence (e.g., “near-live” clinical scenarios), whilst remaining cost-effective and timely for fast version updates (Fig. 1). Previous work demonstrated, the total cost for such a simulation was as little as 2750 USD, including set-up, subject and personnel cost^85^. A recent cost-effective analysis suggested that introducing simulation into a product development lifecycle could lead to cost savings of 37–79%^86^. Other advantages include: scalability^19^, flexibility in design of studies (e.g. different scenarios, various types of participants), feasibility in being implemented as remote and/or distributed^87^, and ability to collect behavioral and/or cognitive metrics. Sophisticated approaches and equipment can be employed, such as eye-tracker analysis or measurement of EEG, which would not be possible in real clinical practice. Furthermore, clinical simulation may also be helpful in facilitating patient engagement and/or Patient and Public Involvement and Engagement (PPIE), an initiative aiming to involving patients and/or representatives from relevant public bodies in the research^88^.

Clinical simulation has been increasingly used in evaluating digital health solutions, including five studies in Table 4, and a further twenty studies from ITX-lab evaluating clinical information systems^89^. For example, in one study^90^ primary care physicians interacted with videoclips of professional patient actors providing standardized responses to clinical scenarios and utilized a CDS tool of clinical prediction rules via an EMR system. In another recently published study^91^, cognitive load and performance of physicians was evaluated for different conditions by randomly assigning participants to baseline EHR (control) or enhanced EHR (simulated environment with features such as automatic sorting and decision support instructions). Moreover, a recent interview study of 10+ companies reported that they found this approach feasible for evidence generation for their own digital solution^19^.

Several academic centers have established clinical simulation test environments, including: The School of Health Information Science (University of Victoria); The Department of Development and Planning (Aalborg University); The IT Experimentarium (ITX) lab (Danish Institute for Medical Simulation)^84^; and, The Institute of Global Health Innovation (IGHI) (Imperial Colleague London)^92^. Indeed, researchers from IGHI have established a simulation test bed specifically to explore application to test digital health solutions. Initial work evaluated the impact of a digital solution on the conduction of cancer multidisciplinary team (MDT) meetings. 56 healthcare professionals (e.g. pulmonologist, oncologists, radiologists, clinical nurse specialists, and thoracic surgeons), who were regular participants at lung cancer tumor boards, were recruited to take 10 simulated MDT sessions. High-fidelity mock patient cases were developed by the study team and clinical experts^93^. Participants discussed up to 10 patient cases, using a standard UK approach to conduct MDTs (paper handout and PACS system) in the control condition, compared with the NAVIFY Tumor Board solution. A manuscript detailing the learnings and results from this pioneer work is under development.

Whilst clinical simulation offers opportunities to prospectively test a digital solution quickly, safely and cost-effectively prior to implementation, there are a few limitations in its use. First, high-fidelity is a prerequisite for generating valid and effective evidence. Therefore, researchers should take efforts to create scenarios representing real clinical practice, recruit the most representative end-users as participants, and provide comprehensive trainings of the digital solutions to the participants before their simulation sessions. Second, while the regulatory space evolves fast, we think clinical simulation results itself alone probably are not adequate for approval application from Health authorities, particularly for higher-risk group of digital solutions that would need to be approved as SaMD. Nevertheless, in these cases, clinical simulations can help to provide initial insights for product development, reduce safety risk for patients, and guide the design of large-scale real clinical studies. Third, for digital solutions that are already adopted in clinical practice, leveraging real-word data (RWD) is probably more suitable. RWD studies could be systematically employed to undertake near real-time evaluation during pilot implementation and post-market monitoring. Indeed, studies utilizing real-world data (RWD) have been encouraged to support regulatory decision making (e.g. The 21st Century Cures Act; Table 3); have been used for clinical evidence generation (e.g. diagnostic and treatment patterns)^94–96^; and can demonstrate solution utility (e.g. meta-data associated with solution features and functionalities).

Finally, we believe clinical simulation can be employed in combination with traditional study designs, e.g., individual-randomization, cluster-level randomization, and micro-randomization to examine different types of digital solutions. For example, clinical simulation-based study with micro-randomization design can be a powerful and pragmatic approach to evaluate the digital solutions with multiple components at early stage of the product development.

## Conclusion

Innovators face significant challenges to overcome the “no evidence, no implementation—no implementation, no evidence” paradox in digital health. We believe that innovative approaches, such as simulation-based research, can enable the generation of higher-quality, lower-cost, and more timely evidence. By considering such methods, end-users will encourage developers to undertake research activities, rather than be intimidated by the complexity, cost, and duration of traditional approaches.

## Data Availability

All data supporting the findings of this study are available within the paper.
